# Common colorectal cancer risk alleles contribute to the multiple colorectal adenoma phenotype, but do not influence colonic polyposis in FAP

**DOI:** 10.1038/ejhg.2014.74

**Published:** 2014-05-07

**Authors:** Timothy H T Cheng, Maggie Gorman, Lynn Martin, Ella Barclay, Graham Casey, Colon Cancer Family Registry, Polly A Newcomb, Graham Casey, David V Conti, Fred Schumacher, Steve Gallinger, Noralane M Lindor, John Hopper, Mark Jenkins, CGEMS, David J Hunter, Peter Kraft, Kevin B Jacobs, David G Cox, Meredith Yeager, Susan E Hankinson, Sholom Wacholder, Zhaoming Wang, Robert Welch, Amy Hutchinson, Junwen Wang, Kai Yu, Nilanjan Chatterjee, Nick Orr, Walter C Willett, Graham A Colditz, Regina G Ziegler, Christine D Berg, Saundra S Buys, Catherine A McCarty, Heather Spencer Feigelson, Eugenia E Calle, Michael J Thun, Richard B Hayes, Margaret Tucker, Daniela S Gerhard, Joseph F Fraumeni, Robert N Hoover, Gilles Thomas, Stephen J Chanock, Meredith Yeager, Nilanjan Chatterjee, Julia Ciampa, Kevin B Jacobs, Jesus Gonzalez-Bosquet, Richard B Hayes, Peter Kraft, Sholom Wacholder, Nick Orr, Sonja Berndt, Kai Yu, Amy Hutchinson, Zhaoming Wang, Laufey Amundadottir, Heather Spencer Feigelson, Michael J Thun, W Ryan Diver, Demetrius Albanes, Jarmo Virtamo, Stephanie Weinstein, Fredrick R Schumacher, Geraldine Cancel-Tassin, Olivier Cussenot, Antoine Valeri, Gerald L Andriole, E David Crawford, Christopher A Haiman, Brian Henderson, Laurence Kolonel, Loic Le Marchand, Afshan Siddiq, Elio Riboli, Timothy J Key, Rudolf Kaaks, William Isaacs, Sarah Isaacs, Kathleen E Wiley, Henrik Gronberg, Fredrik Wiklund, Pär Stattin, Jianfeng Xu, S Lilly Zheng, Jielin Sun, Lars J Vatten, Kristian Hveem, Merethe Kumle, Margaret Tucker, Daniela S Gerhard, Robert N Hoover, Joseph F Fraumeni, David J Hunter, Gilles Thomas, Stephen J Chanock, Mark P Purdue, Mattias Johansson, Diana Zelenika, Jorge R Toro, Ghislaine Scelo, Lee E Moore, Egor Prokhortchouk, Xifeng Wu, Lambertus A Kiemeney, Valerie Gaborieau, Kevin B Jacobs, Wong-Ho Chow, David Zaridze, Vsevolod Matveev, Jan Lubinski, Joanna Trubicka, Neonila Szeszenia-Dabrowska, Jolanta Lissowska, Péter Rudnai, Eleonora Fabianova, Alexandru Bucur, Vladimir Bencko, Lenka Foretova, Vladimir Janout, Paolo Boffetta, Joanne S Colt, Faith G Davis, Kendra L Schwartz, Rosamonde E Banks, Peter J Selby, Patricia Harnden, Christine D Berg, Ann W Hsing, Robert L Grubb III, Heiner Boeing, Paolo Vineis, Françoise Clavel-Chapelon, Domenico Palli, Rosario Tumino, Vittorio Krogh, Salvatore Panico, Eric J Duell, José Ramón Quirós, Maria-José Sanchez, Carmen Navarro, Eva Ardanaz, Miren Dorronsoro, Kay-Tee Khaw, Naomi E Allen, H Bas Bueno-de-Mesquita, Petra H M Peeters, Dimitrios Trichopoulos, Jakob Linseisen, Börje Ljungberg, Kim Overvad, Anne Tjønneland, Isabelle Romieu, Elio Riboli, Anush Mukeria, Oxana Shangina, Victoria L Stevens, Michael J Thun, W Ryan Diver, Susan M Gapstur, Paul D Pharoah, Douglas F Easton, Demetrius Albanes, Stephanie J Weinstein, Jarmo Virtamo, Lars Vatten, Kristian Hveem, Inger Njølstad, Grethe S Tell, Camilla Stoltenberg, Rajiv Kumar, Kvetoslava Koppova, Olivier Cussenot, Simone Benhamou, Egbert Oosterwijk, Sita H Vermeulen, Katja K H Aben, Saskia L van der Marel, Yuanqing Ye, Christopher G Wood, Xia Pu, Alexander M Mazur, Eugenia S Boulygina, Nikolai N Chekanov, Mario Foglio, Doris Lechner, Ivo Gut, Simon Heath, Hélène Blanche, Amy Hutchinson, Gilles Thomas, Zhaoming Wang, Meredith Yeager, Joseph F Fraumeni, Konstantin G Skryabin, James D McKay, Nathaniel Rothman, Stephen J Chanock, Mark Lathrop, Paul Brennan, Brian Saunders, Huw Thomas, Sue Clark, Ian Tomlinson

**Affiliations:** 1 Molecular and Population Genetics Laboratory, Nuffield Department of Clinical Medicine, Wellcome Trust Centre for Human Genetics, Oxford, UK; 2 Department of Preventive Medicine, Keck School of Medicine, University of Southern California, Los Angeles, USA; 3 Wolfson Unit for Endoscopy, Imperial College School of Medicine, St Mark's Hospital, Harrow, UK; 4 Family Cancer Registry, Imperial College School of Medicine, St Mark's Hospital, Harrow, UK; 5 Polyposis Registry, Imperial College School of Medicine, St Mark's Hospital, Harrow, UK; 6 Oxford NIHR Comprehensive Biomedical Research Centre, Wellcome Trust Centre for Human Genetics, Oxford, UK

## Abstract

The presence of multiple (5–100) colorectal adenomas suggests an inherited predisposition, but the genetic aetiology of this phenotype is undetermined if patients test negative for Mendelian polyposis syndromes such as familial adenomatous polyposis (FAP) and *MUTYH-*associated polyposis (MAP). We investigated whether 18 common colorectal cancer (CRC) predisposition single-nucleotide polymorphisms (SNPs) could help to explain some cases with multiple adenomas who phenocopied FAP or MAP, but had no pathogenic *APC* or *MUTYH* variant. No multiple adenoma case had an outlying number of CRC SNP risk alleles, but multiple adenoma patients did have a significantly higher number of risk alleles than population controls (*P*=5.7 × 10^−7^). The association was stronger in those with ≥10 adenomas. The CRC SNPs accounted for 4.3% of the variation in multiple adenoma risk, with three SNPs (rs6983267, rs10795668, rs3802842) explaining 3.0% of the variation. In FAP patients, the CRC risk score did not differ significantly from the controls, as we expected given the overwhelming effect of pathogenic germline *APC* variants on the phenotype of these cases. More unexpectedly, we found no evidence that the CRC SNPs act as modifier genes for the number of colorectal adenomas in FAP patients. In conclusion, common colorectal tumour risk alleles contribute to the development of multiple adenomas in patients without pathogenic germline *APC* or *MUTYH* variants. This phenotype may have ‘polygenic' or monogenic origins. The risk of CRC in relatives of multiple adenoma cases is probably much lower for cases with polygenic disease, and this should be taken into account when counselling such patients.

## Introduction

Most colorectal cancers (CRCs) probably arise from adenomatous polyps. There is a clinically important subset of patients who are found to have multiple (five or more) colorectal adenomas, a phenotype that is suggestive of an inherited genetic predisposition. Identifying the genetic basis of these patients' tumours is clinically informative in terms of determining the natural history of their disease, the cancer risks in relatives and the optimal means of surveillance and early treatment.

Some patients with ≥5 adenomas have one of the rare Mendelian polyposis syndromes, such as classical or attenuated familial adenomatous polyposis (FAP),^1^ *MUTYH*-associated polyposis (MAP)^2^ or polymerase proofreading-associated polyposis (PPAP).^3^ The number of adenomas is highly variable in each syndrome, and for FAP, the underlying causes of this phenotypic variation have been studied in some detail. FAP is caused by germline *APC* variants and the position of the variant explains some of the variation in polyp numbers. For example, although FAP patients usually develop hundreds or thousands of adenomas, a few individuals with variants in proximal, distal or alternatively spliced regions of the gene have so-called attenuated disease, with tens or fewer tumours.^4^ The position of the germline *APC* variants also influences the ‘classical' (>100 adenomas) FAP phenotype: for example, severe colonic polyposis is associated with germline variants near codon 1309.^5^ However, several studies have also addressed the possibility that modifier genes unlinked to *APC* influence the FAP phenotype.^6, 7, 8^

Only a minority of individuals with 5–20 colorectal adenomas test positive for any pathogenic germline variant^9^ and the set of patients with multiple colorectal adenomas is genetically heterogeneous. Recently, genome-wide association studies (GWAS) have identified haplotype-tagging single-nucleotide polymorphisms (SNPs) that are associated with CRC risk in the general population.^10^ Some of these variants are close to loci (eg, *GREM1*, *BMP2*, *BMP4*, *POLD3* and *MYC*) that are functionally related to the genes involved in the Mendelian polyposis syndromes. Each SNP has a modest effect size (typically 10–20% increased risk per allele). By testing individuals with small numbers of colorectal adenomas (median=1, interquartile range=1–2), but no history of CRC, we previously showed that some SNPs predispose to CRC through the development of adenomas.^11^

In this study, we have examined whether the multiple adenoma phenotype can be explained in some cases by common risk alleles of individually modest effects. We have also assessed whether the common CRC predisposition SNPs influence the severity of colonic polyposis in FAP.

## Materials and Methods

Genomic DNA was extracted from peripheral blood of 178 unrelated individuals with multiple (5–100) adenomatous polyps (median=10, IQR=7–17) at first colonoscopy based on routine histopathological reports (Supplementary Figure 1). Seventy-one patients had CRC at presentation or subsequently (Table 1), or had a family history of CRC. No case had any polyp of hamartomatous morphology. A number had several serrated polyps, but in all cases, the classical adenoma was the majority morphology. Patients were from throughout the United Kingdom and all were of northern European ancestry. Patients were not selected on the basis of age, adenoma size or specific histology. Median age of presentation was 55 years (IQR=48–64) and 34% of patients were female. Adenoma number was not associated with increased patient age (*P*=0.97) or with gender (*P*=0.32). FAP, MAP and PPAP had been excluded in each case using direct sequencing of (i) the regions of *APC* associated with attenuated disease (and the entire gene for those with close to 100 adenomas), (ii) the common northern European *MUTYH* variants p.Tyr179Cys and p.Gly396Asp^12^ and (iii) the exonuclease domains of *POLE* and *POLD1*.^3^ One hundred and forty-two patients (79 families) with FAP and pathogenic germline *APC* variants were also studied, as were 30 cases with a classical FAP phenotype and no identified disease-causing variant in *APC*, *MUYTH*, *POLE* or *POLD1*. Data on age, sex and number of polyps at colectomy from the FAP patients were obtained from the St Mark's Hospital Polyposis Registry, Harrow, UK.

For the multiple adenoma and FAP patients, we used KASPar assays (KBiosciences, Hertfordshire, UK) to genotype 18 published CRC SNPs (rs6691170 chr1.hg19:g.220112069G>T, rs6687758 chr1.hg19:g.220231571A>G, rs10936599 chr3.hg19:g.170974795 C>T, rs16892766 chr8.hg19:g.117699864 A>C, rs6983267 chr8.hg19:g.128482487 G>T, rs10795668 chr10.hg19:g.8741225 G>A, rs3802842 chr11.hg19:g.110676919 A>C, rs7136702 chr12.hg19:g.49166483 C>T, rs11169552 chr12.hg19:g.49441930 C>T, rs4444235 chr14.hg19:g.53480669 T>C, rs1957636 chr14.hg19:g.53629768 G>A, rs4779584 chr15.hg19:g.30782048 C>T, rs9929218 chr16.hg19:g.67378447 G>A, rs4939827 chr18.hg19:g.44707461 T>C, rs10411210 chr19.hg19:g.38224140 C>T, rs961253 chr20.hg19:g.6352281 C>A, rs4813802 chr20.hg19:g.6647595 T>G and rs4925386 chr20.hg19:g.60354439 C>T). All of these assays had previously been validated as showing >98% genotype concordance with Illumina SNP array genotypes as part of the CRC GWAS. Eight samples were excluded for having individual SNP genotype call rates of <94%. The overall genotyping call rate was >99.5%. None of the markers showed significant deviation from Hardy–Weinberg equilibrium (*P*>0.05).

As controls, to avoid overlap with the published CRC GWAS, we extracted SNP genotypes for the 18 SNPs from cancer-free individuals within the publicly available Colorectal Cancer Family Registry (CFR) (http://coloncfr.org/) and CGEMS (http://dceg.cancer.gov/research/how-we-study/genomic-studies/cgems-summary) data sets that had been genotyped using Illumina genome-wide tagSNP arrays. To control for population stratification, we conducted principal component analysis to ensure that the CFR samples used clustered with UK population individuals of northern European ancestry (Supplementary Figure 2).

**Figure 1:**
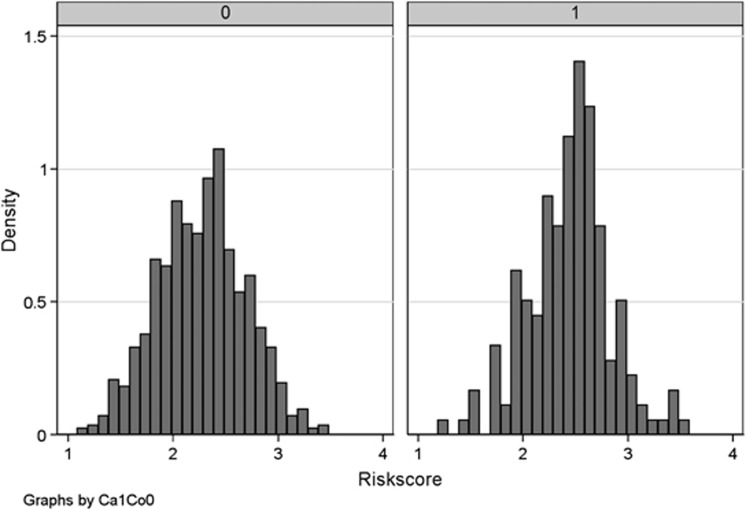
Histograms comparing the risk score distribution in multiple adenoma cases (1) and controls (0). A simple count of an individual's total number of high-risk CRC alleles, without taking differing SNP effect sizes into account, yielded similar results.

We calculated for each individual a SNP risk score defined by


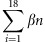


where *β* is the ln(odds ratio (OR)) (>0) for each SNP derived from unconditional logistic regression analysis in CFR cases and CGEMS controls (Supplementary Tables 1 and 2 and Figure 1), and *n* is the number of risk alleles (0–2) carried by that individual. This resulted in a theoretical range of risk scores between 0 and 4.78, where the minimum and maximum score would represent individuals homozygous for all the protective and risk alleles respectively.

## Results and discussion

The SNP risk score was approximately normally distributed in both adenoma cases and controls (Shapiro–Wilk test, *P*>0.13). We initially wondered whether any of the cases had an outlying number of CRC SNP risk alleles. The maximum number of risk alleles carried by any patient was 26/36, equivalent to a risk score of 3.53. Four cases and three controls had risk scores of over 3.40. These data suggested that the risk score had limited use as a predictor of multiple adenomas on an individual basis. This result was not unexpected, given that the known CRC SNPs account for only a minority of adenoma risk.^11^

As a more general test of the hypothesis that common risk alleles contribute to the multiple adenoma phenotype, we compared the SNP risk score distributions in multiple adenoma cases and controls. There was a significantly higher risk score (*P*=5.8 × 10^−7^, *t*-test; Table 1 and Figure 1) in multiple adenoma cases (mean=2.44, SD=0.40) than controls (mean=2.27, SD=0.42). The association remained present (*P*=0.0011) when the analysis was restricted to the 107 cases with no known personal or family history of CRC (mean score=2.41, SD=0.40). Of the 178 multiple adenoma cases, 103 had ≥10 adenomas and 75 had 5–9 adenomas. Both case groups individually had significantly higher risk scores than controls (Table 1 and Figure 1), but in the group with ≥10 adenomas, the mean risk score was higher (2.48) than that in the patients with 5–9 adenomas (2.39). Ordered logistic regression analysis on the three groups (10+ adenomas *vs* 5–9 adenomas *vs* population controls) showed that the risk score was correlated with adenoma numbers (*P*=9.5 × 10^−7^).

The 18 CRC SNPs explained 4.3% of the variance in the risk of multiple adenomas. Using multivariate logistic regression to assess specific polymorphisms, we found that three individual SNPs, rs6983267 (OR=1.54, 95% CI: 1.21–1.97, *P*=3.74 × 10^−4^), rs10795668 (OR=0.64, 95% CI: 0.49–0.84, *P*=0.00148) and rs3802842 (OR=1.31, 95% CI: 1.03–1.69, *P*=0.0278) were nominally significantly associated with the multiple adenoma phenotype. These three SNPs alone explained ∼3% of the variance in multiple adenoma risk.

Of note, Hes *et al*^13^ recently reported associations between individual CRC SNPs and adenoma risk in a similar data set to ours, although no risk score was calculated. Hes *et al*^13^ found rs3802842 to be the SNP most significantly associated with adenoma risk, with additional evidence for associations with rs6983267 and with a further SNP, rs4779584. In our previous analysis of patients with smaller numbers of adenomas, rs3802842 and rs6983267 again showed strong associations with risk, although rs4779584 did not. For rs10795668, the evidence for an association with rs10795668 was only moderate in the Hes *et al* data^13^ and our own previous study,^11^ but the direction and magnitude of effect were consistent in all three studies. Our other four previously reported adenoma SNPs (rs10936599, rs4444235, rs1957636, rs4939827, rs961253)^11^ were not as well supported by this study or by Hes *et al*^13^ (details not shown), although this might have resulted from the relatively small sizes and low power of the two multiple adenoma case collections.

Our set of 142 FAP patients with germline *APC* variants had a very similar risk score distribution to the controls, as did the 30 cases with classical FAP and no identified pathogenic variant in the polyposis genes (*P*=0.53 and 0.42 respectively; Table 1). Given the overwhelming effect of the germline *APC* variant on the FAP phenotype, the former result was not unexpected, and provided reassurance that the controls used were representative of the general UK population.

The severity of colorectal polyposis in our FAP cases (number of colorectal adenomas at prophylactic colectomy) was known for 64 patients from 30 families with pathogenic germline *APC* variants. Including sex, age and the position of the germline *APC* variant (codons 1265–1389 *vs* regions associated with attenuated FAP *vs* other)^14^ as covariates in a linear regression analysis, we tested whether the CRC SNPs were associated with FAP severity. Although *APC* variant position was strongly associated with polyp number (details not shown), there was no association with any other variable. In particular, SNP risk score showed very little evidence of a positive association with polyp number (OR=0.94, 95% CI: 0.87–1.01, *P*=0.09), and no individual SNP was nominally associated with polyp count.

In general, the phenotypic overlap between Mendelian and ‘sporadic' disease is particularly important for the common cancers, in terms of the clinical management of cancer families and/or those with multiple tumours. Our results probably underestimate the effects of the CRC SNPs on the multiple adenoma phenotype, because the publicly available population controls were not known to be adenoma-free; furthermore, there almost certainly exist undiscovered, common CRC risk variants. A further consideration is that a small number of our multiple adenomas cases may have carried germline variants in the mismatch repair genes (*MSH2*, *MLH1*, *MSH6*, *PMS2*) or the juvenile polyposis genes (*SMAD4*, *BMPR1A*). We were a little surprised to find no evidence that the CRC SNPs affected the severity of the colorectal phenotype in FAP cases, because adenoma pathogenesis is often thought to be similar in FAP and sporadic lesions. Genotyping of modifier genes could influence the management of FAP patients – for example, in choosing between ileorectal anastomosis and pouch formation. However, our data suggest that the known CRC SNPs cannot be used for this purpose.

In conclusion, although unidentified Mendelian predisposition genes for multiple adenomas may exist, we have shown that common CRC risk variants are likely to contribute to the multiple adenoma phenotype. It is highly plausible that some of the multiple adenoma cases carry additional, unknown susceptibility variants with moderate or small effects. Multiple adenoma cases in whom the known Mendelian syndromes have been excluded are often not clinically distinguishable from the Mendelian conditions of attenuated FAP, MAP or PPAP. However, some of the multiple adenoma patients will have ‘polygenic' rather than monogenic disease, with an accompanying lower risk of CRC in family members. Although polygenic multiple adenoma cases cannot currently be identified positively by genetic testing, the existence of non-Mendelian genetic adenoma aetiology should be recognised when counselling the families of multiple adenoma patients and monitoring the screening regimens of at-risk relatives.

## Figures and Tables

**Table 1:** Summary risk score statistics for each group

	n	*Mean risk score*	*Standard deviation*	t	P-*value*
MA	178	2.44	0.40	5.12	5.78 × 10^−7^
MA no history CRC	107	2.41	0.40	3.28	0.0011
MA (≥10)	103	2.48	0.39	4.44	1.98 × 10^−5^
MA (5–9)	75	2.39	0.40	2.08	0.041
FAP-like, APC wild type	30	2.21	0.41	0.83	0.42
FAP, APC mutant	142	2.25	0.42	0.63	0.53
CFR controls	818	2.27	0.42	—	—

MA=all multiple adenoma cases; MA no history CRC=MA cases with no personal or family history of CRC; MA (≥10)=MA cases with 10 or more adenomas; MA (5–9)=M cases with 5–9 adenomas.

*t*-Test results are shown compared with the control group. All sample sets had normally distributed risk scores (*P*>0.05, Shapiro–Wilk test).
