# Supplementary material for: Common colorectal cancer risk alleles contribute to the multiple colorectal adenoma phenotype, but do not influence colonic polyposis in FAP
Source: Eur J Hum Genet. 2014 May 7;23(2):260–3. doi: 10.1038/ejhg.2014.74 (PMC4140766; doi:10.1038/ejhg.2014.74)

Supplementary material

Supplementary Table 1: Effect sizes of 18 CRC GWAS common variants based on CFR cases and CGEMS controls

Since the discovery phase of GWAS tends to overestimate the effect size of the risk alleles, we wished to obtain an independent measure of effect size. We therefore determined β for each SNP from an analysis of 912 colorectal cancer cases (colon CFR) and 3,160 cancer-free controls (CGEMS study) that had not previously been used in any published CRC GWAS discovery phase; CFR controls were not used because they had already been chosen for making comparisons with our multiple adenoma cases. We found that all βs determined from the CFR-CGEMS association analysis were in the same direction as published CRC GWAS.

SNP	Chr	Build 19 Pos	Minor	Other	Risk	MAF MA	MAF controls	OR	L95	U95	β	SE	P
rs6691170	1	220,112,069	T	G	T	0.37	0.37	1.00	0.85	1.13	0.00	0.07	$9.75x10^{-1}$
rs6687758	1	220,231,571	G	A	G	0.19	0.19	1.01	0.75	1.04	0.01	0.08	$9.32x10^{-1}$
rs10936599	3	170,974,795	T	C	C	0.24	0.25	0.92	0.80	1.10	-0.08	0.08	$1.95x10^{-1}$
rs16892766	8	117,699,864	C	A	C	0.09	0.08	1.17	0.96	1.55	0.16	0.12	$8.90x10^{-2}$
rs6983267	8	128,482,487	T	G	G	0.43	0.49	0.79	0.67	0.89	-0.25	0.07	$8.26x10^{-6}$
rs10795668	10	8,741,225	A	G	G	0.30	0.33	0.86	0.74	0.99	-0.15	0.07	$1.11x10^{-2}$
rs3802842	11	110,676,919	C	A	C	0.34	0.30	1.17	1.12	1.50	0.16	0.07	$4.77x10^{-3}$
rs7136702	12	49,166,483	T	C	T	0.39	0.35	1.16	1.11	1.49	0.15	0.07	$5.80x10^{-3}$
rs11169552	12	49,441,930	T	C	C	0.25	0.26	0.98	0.77	1.04	-0.02	0.08	$7.56x10^{-1}$
rs4444235	14	53,480,669	C	T	C	0.49	0.46	1.14	0.94	1.24	0.13	0.07	$1.12x10^{-2}$
rs1957636	14	53,629,768	A	G	A	0.44	0.40	1.14	1.04	1.38	0.13	0.07	$1.24x10^{-2}$
rs4779584	15	30,782,048	T	C	T	0.22	0.19	1.17	0.98	1.38	0.16	0.09	$1.86x10^{-2}$
rs9929218	16	67,378,447	A	G	G	0.27	0.29	0.93	0.79	1.07	-0.07	0.08	$2.15x10^{-1}$
rs4939827	18	44,707,461	C	T	T	0.42	0.50	0.73	0.66	0.87	-0.31	0.07	$6.83x10^{-9}$
rs10411210	19	38,224,140	T	C	C	0.08	0.10	0.86	0.77	1.26	-0.15	0.13	$1.07x10^{-1}$
rs961253	20	6,352,281	A	C	A	0.38	0.36	1.07	0.99	1.32	0.07	0.07	$2.19x10^{-1}$
rs4813802	20	6,647,595	G	T	G	0.39	0.35	1.21	1.09	1.43	0.19	0.07	$4.43x10^{-4}$
rs4925386	20	60,354,439	T	C	C	0.28	0.32	0.82	0.80	1.07	-0.20	0.08	$7.92x10^{-4}$

Supplementary Table 2. Individual SNP genotypes for each case studied.

MA=multiple adenoma patient; FAP=FAP patient

Genotypes are shown as total minor allele count (i.e. 0=major allele homozygote, 1=heterozygote, 2=minor allele homozygote)

ID	rs6691170	rs6687758	rs10936599	rs16892766	rs6983267	rs10795668	rs3802842	rs7136702	rs11169552	rs4444235	rs1957636	rs4779584	rs9929218	rs4939827	rs10411210	rs961253	rs4813802	rs4925386
MA1_A01	2	1	2	0	2	1	0	0	1	1	1	1	2	1	2	1	1	1
MA1_A03	0	0	2	0	1	2	0	0	1	1	2	0	0	0	1	1	1	2
MA1_A04	1	1	2	0	1	1	0	0	1	1	0	1	2	2	2	1	1	1
MA1_A05	1	2	2	1	1	2	0	0	0	2	0	0	2	1	1	1	1	2
MA1_A06	1	0	1	0	2	1	0	1	1	0	0	0	1	1	2	1	0	1
MA1_A07	2	2	2	0	2	2	1	2	2	0	0	0	2	0	2	1	0	0
MA1_A08	2	2	2	0	1	2	1	0	1	2	2	0	2	1	2	1	1	1
MA1_A09	1	1	2	0	2	1	1	0	1	0	0	1	2	2	2	1	0	1
MA1_A10	2	2	2	0	2	2	0	2	2	1	1	1	1	1	2	0	1	2
MA1_A11	1	0	1	0	1	2	0	2	2	1	1	2	2	1	1	1	0	2
MA1_A12	1	1	2	0	2	2	0	0	0	1	1	0	1	1	2	2	0	2
MA1_B01	1	0	0	0	0	1	2	0	1	1	1	0	2	2	2	1	2	2
MA1_B02	0	0	2	0	1	2	1	0	1	1	1	2	0	1	2	2	0	1
MA1_B03	0	0	2	0	1	1	0	2	1	0	1	1	2	2	2	1	0	1
MA1_B04	0	0	2	0	2	1	1	1	2	0	0	0	2	1	2	1	0	1
MA1_B05	0	0	1	0	1	1	1	0	0	2	1	1	1	2	2	0	1	1
MA1_B06	1	0	1	0	1	2	0	1	2	1	1	0	2	2	1	0	1	1
MA1_B07	2	0	1	0	2	1	1	0	1	1	1	1	1	1	2	0	1	2
MA1_B08	2	0	2	0	2	2	0	1	1	0	1	0	2	1	2	1	0	2
MA1_B09	1	0	1	0	0	1	1	0	1	0	1	0	1	2	2	0	1	0
MA1_B10	1	0	2	0	2	2	1	0	1	1	0	1	1	2	2	0	0	1
MA1_B11	2	2	2	0	2	2	0	1	1	2	0	2	2	1	2	0	0	0
MA1_B12	1	0	2	0	2	2	1	1	2	1	1	0	2	0	2	1	1	1
MA1_C01	1	1	0	0	1	1	1	1	2	1	0	0	1	1	2	1	0	2
MA1_C02	0	1	0	0	0	2	2	1	2	0	2	0	2	2	2	1	1	2
MA1_C03	0	1	2	0	1	1	1	0	1	1	1	0	2	1	0	2	1	2
MA1_C04	1	1	2	0	1	2	0	2	2	1	0	0	2	1	2	0	0	2
MA1_C05	1	0	2	0	1	2	1	2	2	1	0	0	2	0	2	1	0	1
MA1_C06	1	0	1	1	2	2	2	1	1	1	1	1	2	2	2	1	0	2
MA1_C07	1	1	1	0	2	2	0	2	1	1	2	0	2	2	1	1	1	1
MA1_C08	1	1	2	1	1	2	1	1	1	1	0	0	2	1	1	1	0	1
MA1_C09	1	1	2	2	2	1	1	2	2	1	0	2	1	1	2	0	0	2
MA1_C10	1	0	2	1	0	2	1	1	2	2	1	0	2	1	2	2	0	2
MA1_C11	2	1	1	0	1	2	1	1	2	0	0	0	1	1	2	1	1	2
MA1_C12	1	0	0	0	2	1	0	2	2	2	1	0	1	0	2	1	0	1
MA1_D01	1	1	2	1	2	2	0	0	2	1	1	0	1	2	2	1	2	1
MA1_D02	2	1	1	0	2	2	0	0	2	0	0	2	1	1	2	1	1	1
MA1_D03	0	0	1	0	0	1	2	0	1	1	1	0	2	0	2	1	0	2
MA1_D04	1	0	1	0	1	1	2	2	2	0	0	0	1	2	2	0	2	1
MA1_D05	1	1	2	0	2	1	2	1	2	2	0	0	1	1	2	1	1	1
MA1_D06	1	1	1	1	1	2	0	2	2	1	1	0	1	1	2	1	2	1
MA1_D07	0	0	2	0	0	2	0	1	1	0	1	0	2	2	2	1	2	2
MA1_D08	1	0	1	1	1	2	1	0	1	2	1	1	2	2	2	2	1	2
MA1_D09	0	0	1	0	1	1	1	0	1	1	2	1	1	2	2	1	2	1

MA1_D10	2	1	2	0	1	2	0	1	2	1	1	0	2	2	1	0	1	2
MA1_D11	1	1	1	0	1	1	1	0	1	2	2	1	2	1	0	1	0	2
MA1_D12	0	0	1	1	0	1	1	2	2	1	1	2	1	1	2	1	0	2
MA1_E01	0	0	2	0	1	2	1	0	2	2	0	0	0	1	2	1	0	2
MA1_E02	0	0	2	0	1	2	1	0	1	0	0	1	1	0	2	1	2	2
MA1_E03	1	0	1	2	2	2	1	1	1	0	1	0	2	1	2	2	0	2
MA1_E04	1	0	1	0	0	2	0	2	2	1	1	0	2	1	1	0	0	1
MA1_E05	1	1	1	0	1	2	0	2	1	1	1	0	2	2	2	0	0	1
MA1_E06	1	1	1	0	1	1	0	0	1	1	2	0	2	1	2	0	0	2
MA1_E07	0	0	2	0	1	2	1	0	0	0	0	0	1	0	2	2	0	2
MA1_E08	1	0	2	0	2	1	1	1	2	0	1	0	1	2	1	1	1	1
MA1_E09	1	0	2	1	2	2	0	0	2	1	1	0	1	1	2	1	0	2
MA1_E11	1	1	2	0	2	2	2	0	2	1	1	0	2	1	2	0	1	2
MA1_E12	2	0	2	0	1	2	1	1	1	1	0	0	2	1	2	0	1	2
MA1_F01	0	0	2	0	0	0	0	2	2	1	1	0	2	2	2	0	1	2
MA1_F02	0	0	2	0	0	2	0	1	2	1	2	0	2	1	2	1	1	2
MA1_F03	0	0	2	0	0	1	2	0	2	0	1	0	1	0	2	0	2	1
MA1_F04	0	0	2	0	0	2	1	1	2	1	1	0	1	2	2	2	0	1
MA1_F05	0	1	0	0	2	2	1	1	2	0	0	1	1	1	2	1	1	2
MA1_F06	0	0	2	0	2	1	0	0	2	2	2	1	2	0	2	0	2	0
MA1_F07	0	0	2	0	1	1	1	0	2	1	2	1	1	2	2	1	0	1
MA1_F08	1	0	2	0	1	2	0	1	2	1	1	0	2	2	2	1	0	1
MA1_F09	2	0	2	0	1	2	0	0	1	1	1	0	0	2	2	1	0	2
MA1_F10	1	1	2	0	1	0	0	0	2	1	2	0	1	1	2	0	1	2
MA1_F11	0	1	2	0	2	2	1	1	0	1	1	0	2	1	2	0	0	2
MA1_G01	1	0	1	0	1	1	0	0	1	2	1	0	2	1	2	2	0	2
MA1_G02	0	0	1	0	1	1	2	1	2	2	1	0	2	2	1	1	0	1
MA1_G03	0	0	2	0	1	1	1	1	2	2	1	1	1	0	2	1	1	2
MA1_G04	1	1	1	0	1	1	1	1	2	1	0	1	2	2	2	0	1	1
MA1_G05	1	1	2	0	1	2	1	0	1	1	1	1	1	0	2	1	0	1
MA1_G06	1	0	1	0	1	2	2	1	2	0	0	1	2	2	2	1	1	1
MA1_G07	1	2	2	0	2	1	1	1	2	1	0	1	2	0	2	1	0	1
MA1_G08	1	0	0	0	1	0	0	1	1	0	1	0	1	0	1	1	1	1
MA1_G09	1	1	2	0	0	1	0	1	2	1	0	1	2	1	2	1	0	2
MA1_G10	2	1	2	0	2	1	0	0	1	1	2	1	2	2	2	0	1	0
MA1_G11	0	0	2	1	1	1	0	0	2	1	1	0	0	1	2	1	1	1
MA1_H01	1	2	0	0	1	0	0	1	2	2	0	0	2	2	2	0	0	1
MA1_H02	1	1	1	0	0	2	0	0	2	2	1	0	1	1	2	0	2	1
MA1_H03	1	0	1	0	0	1	0	0	2	0	1	0	2	2	2	0	0	0
MA1_H04	0	0	2	0	2	2	0	2	2	1	1	1	2	2	2	1	1	2
MA1_H05	0	0	1	0	2	2	0	0	1	0	2	1	1	2	2	1	0	1
MA1_H06	1	1	1	0	2	2	1	0	2	2	0	1	1	2	1	0	1	2
MA1_H07	1	0	2	0	2	2	0	0	1	2	2	1	1	1	1	0	2	1
MA1_H08	0	0	2	0	2	2	0	0	1	2	2	0	2	1	1	0	1	1
MA1_H10	2	1	0	0	0	1	1	0	0	1	2	1	1	2	2	1	0	2
MA2_A01	1	1	2	0	0	1	0	2	2	1	0	0	2	1	2	0	0	0
MA2_A02	0	1	1	0	0	2	1	0	1	1	1	1	2	0	2	1	0	0
MA2_A03	0	0	2	1	2	1	1	1	2	2	0	0	1	0	2	1	1	2
MA2_A04	0	0	1	0	2	1	0	2	1	1	1	1	2	1	2	1	1	1

MA2_A05	2	1	2	0	1	2	1	1	2	1	1	1	1	0	2	2	1	2
MA2_A06	0	0	1	0	2	2	2	1	2	1	0	1	2	0	2	0	1	0
MA2_A07	1	1	2	0	2	1	0	0	2	1	1	0	1	1	2	0	1	1
MA2_A08	2	1	2	0	1	1	0	1	2	1	1	1	2	1	2	0	2	2
MA2_A09	1	0	2	2	1	2	2	0	1	2	1	0	2	1	1	1	0	2
MA2_A10	0	0	1	0	2	1	0	0	1	1	1	0	2	2	1	1	0	1
MA2_A11	0	0	1	1	1	2	1	1	1	2	2	0	2	1	2	0	1	1
MA2_A12	0	0	2	1	0	2	0	0	0	1	0	0	2	0	2	2	0	2
MA2_B01	1	0	1	0	1	2	1	0	0	1	1	1	2	2	1	2	1	1
MA2_B02	1	1	1	0	2	1	1	0	2	1	0	1	1	0	1	2	2	2
MA2_B03	1	1	1	0	2	1	1	1	1	1	2	0	2	1	2	0	0	2
MA2_B04	1	1	2	0	0	2	0	0	0	1	2	0	2	1	2	0	1	2
MA2_B05	0	0	2	0	1	1	0	0	1	0	0	2	2	0	2	0	2	2
MA2_B06	2	0	2	0	2	2	1	2	2	1	1	1	1	2	2	1	1	2
MA2_B07	0	0	0	0	0	1	0	1	2	0	1	0	2	0	2	1	1	2
MA2_B08	0	1	2	1	1	1	1	1	2	1	2	0	2	1	2	2	0	1
MA2_B09	1	1	2	1	2	1	0	1	2	1	1	0	1	1	2	0	1	1
MA2_B10	0	0	2	1	2	0	0	0	1	1	0	0	2	1	2	0	2	2
MA2_B11	0	0	2	0	1	2	1	0	1	0	0	0	2	1	2	1	1	0
MA2_B12	0	0	2	0	2	2	0	1	2	2	0	0	1	0	2	1	1	1
MA2_C01	0	0	2	0	0	1	1	2	2	0	0	1	1	2	2	1	1	2
MA2_C02	0	1	2	0	2	2	1	0	0	2	1	0	2	1	2	1	0	1
MA2_C03	0	0	2	0	2	2	1	2	2	1	1	1	0	2	0	0	0	0
MA2_C04	0	0	2	0	2	2	1	1	2	0	1	0	1	1	2	0	1	2
MA2_C05	2	0	1	1	2	2	0	1	1	0	0	0	2	1	2	1	1	2
MA2_C06	1	1	2	0	2	2	1	2	2	1	1	1	0	0	2	1	1	0
MA2_C07	1	1	2	0	0	0	1	1	1	2	0	0	2	1	2	1	2	2
MA2_C08	0	0	1	0	2	2	2	2	2	1	1	0	1	0	2	0	1	2
MA2_C09	1	0	2	0	1	1	0	0	1	1	2	0	2	0	2	2	0	1
MA2_C10	1	1	1	0	1	2	0	1	2	2	1	0	2	1	2	0	0	2
MA2_C11	1	0	2	0	1	2	0	0	2	1	1	0	2	0	1	1	1	2
MA2_C12	1	0	2	0	1	1	2	1	2	2	1	0	1	0	2	0	1	2
MA2_D01	1	0	2	0	0	1	1	0	2	0	0	1	1	2	2	1	0	2
MA2_D02	1	1	2	0	1	1	1	1	1	1	0	0	1	1	2	0	2	1
MA2_D03	1	0	2	2	2	1	0	0	1	0	2	0	2	1	2	2	0	0
MA2_D04	1	1	2	0	1	1	0	1	2	1	0	1	1	1	2	2	1	0
MA2_D05	1	0	1	1	0	2	2	0	2	2	1	0	2	0	2	0	1	2
MA2_D06	0	1	1	0	2	2	0	1	1	1	0	2	2	2	2	1	0	2
MA2_D07	0	0	1	0	2	1	1	0	0	1	0	0	2	1	2	0	0	1
MA2_D08	0	0	2	0	2	1	0	0	1	1	1	0	2	1	2	1	2	1
MA2_D09	2	1	1	0	2	2	2	2	2	0	0	0	1	2	2	0	1	1
MA2_D10	0	0	2	1	1	1	0	0	1	2	1	1	2	1	2	2	0	1
MA2_D11	0	0	2	0	1	2	0	0	2	2	1	1	0	1	2	0	0	2
MA2_D12	0	0	2	0	1	2	2	2	2	1	1	0	2	1	2	0	2	1
MA2_E01	0	0	2	1	1	1	1	0	2	1	0	0	1	1	2	0	0	1
MA2_E02	1	1	2	0	2	2	2	2	2	1	2	0	1	1	2	0	2	2
MA2_E03	0	0	2	1	1	2	1	0	0	1	1	0	2	1	2	0	1	1
MA2_E04	0	0	2	0	2	2	0	2	2	1	1	0	1	1	2	1	1	1
MA2_E05	0	0	2	0	1	2	1	1	2	1	1	0	1	2	2	1	2	1

MA2_E06	1	1	2	0	2	2	0	0	1	1	1	1	1	2	2	0	0	1
MA2_E08	1	0	1	0	2	2	0	2	2	0	0	2	1	1	2	2	0	2
MA2_E09	1	2	1	0	1	2	0	1	2	0	1	1	2	1	2	1	1	2
MA2_E10	1	1	1	0	2	2	0	1	1	0	0	2	1	1	1	1	2	2
MA2_E11	2	0	2	0	2	1	1	0	1	2	1	1	1	1	2	1	1	0
MA2_E12	2	1	2	0	2	1	2	1	0	1	1	0	2	0	2	0	0	1
MA2_F01	0	0	1	0	1	2	1	0	1	1	2	1	2	1	2	0	2	1
MA2_F02	0	0	1	0	1	2	1	0	1	2	0	0	2	2	2	0	0	2
MA2_F03	1	1	2	0	2	2	2	0	1	1	2	1	1	0	2	0	0	2
MA2_F04	0	1	2	0	1	2	1	2	2	2	1	0	2	2	2	1	1	1
MA2_F05	1	1	2	1	1	1	0	1	1	2	0	1	1	1	2	1	0	2
MA2_F06	0	0	2	0	2	1	0	1	1	0	0	1	1	0	2	1	0	2
MA2_F07	0	0	1	0	2	2	0	1	2	1	1	1	1	1	2	0	0	2
MA2_F08	1	1	2	0	2	1	1	1	1	0	0	0	1	1	2	1	0	1
MA2_F09	0	0	2	0	1	1	1	0	0	2	1	0	1	2	2	0	2	2
MA2_F10	1	1	2	2	1	1	0	1	2	2	0	0	2	0	2	0	1	1
MA2_F11	1	2	0	0	1	1	1	1	2	2	0	0	0	2	2	0	0	0
MA2_G01	0	0	2	0	2	2	2	0	2	2	0	0	2	1	2	0	1	2
MA2_G02	1	2	1	0	1	2	2	1	2	2	2	1	0	2	2	1	1	1
MA2_G03	2	0	2	0	2	2	0	1	2	0	0	1	2	2	2	0	0	2
MA2_G04	2	0	2	0	2	1	0	1	1	0	1	0	2	1	2	0	0	2
MA2_G05	1	0	1	0	0	2	0	2	2	0	0	0	2	1	2	0	2	2
MA2_G06	2	0	1	0	1	2	0	1	1	2	1	1	2	1	2	2	0	1
MA2_G07	1	0	1	0	1	2	1	1	2	0	0	0	2	1	2	1	2	1
MA2_G08	1	1	1	0	1	2	1	0	1	0	0	0	1	2	2	1	1	1
MA2_G09	0	0	2	0	0	1	0	1	1	0	0	0	2	1	2	1	1	2
MA2_G10	1	0	1	0	1	2	1	1	2	1	0	0	1	2	2	1	1	1
MA2_G11	0	0	1	0	1	1	1	1	1	1	1	0	1	1	2	2	1	2
MA2_G12	0	1	1	0	0	1	1	0	1	2	1	0	2	1	2	1	0	2
MA2_H01	1	1	2	0	2	2	1	0	1	1	2	0	0	1	2	2	1	1
MA2_H02	1	2	1	1	1	0	1	2	2	1	0	0	1	1	2	1	0	0
MA2_H03	1	1	2	0	2	1	1	1	1	0	1	1	1	2	2	1	2	1
MA2_H04	0	0	2	0	1	2	1	2	2	0	1	0	1	1	2	2	1	1
MA2_H05	1	1	2	0	0	1	1	1	1	1	1	0	2	1	2	1	0	1
MA2_H06	1	1	1	0	1	2	1	0	2	2	2	0	1	1	2	0	1	2
MA2_H07	1	2	2	0	1	1	1	1	1	0	1	0	2	1	2	0	1	1
FAP1_A01	1	1	1	0	0	2	0	1	2	1	0	0	1	2	2	2	0	2
FAP1_A02	1	0	2	1	2	1	0	2	1	2	0	0	0	0	2	1	0	1
FAP1_A03	1	1	2	0	1	1	0	1	1	1	1	1	1	2	2	0	1	2
FAP1_A04	0	0	1	0	1	1	0	1	2	2	2	1	1	1	2	2	2	1
FAP1_A05	1	1	2	0	0	2	0	2	2	1	0	0	2	2	1	0	1	1
FAP1_A06	1	0	1	0	2	1	1	1	2	1	0	1	2	0	2	1	0	1
FAP1_A07	0	0	2	0	1	2	0	0	1	1	1	0	2	1	2	1	1	1
FAP1_A08	0	0	0	0	1	2	1	1	1	0	1	1	2	1	2	1	1	1
FAP1_A10	1	0	2	1	0	2	1	1	1	1	0	0	2	2	1	0	1	1
FAP1_A11	1	1	2	0	1	2	0	0	0	1	1	1	1	2	1	0	1	2
FAP1_A12	1	1	2	0	1	1	0	0	2	0	0	1	2	1	2	0	0	1
FAP1_B01	0	0	2	1	0	0	0	0	2	2	0	0	1	2	2	0	1	2
FAP1_B02	1	0	2	1	1	1	0	2	1	1	0	1	0	0	0	1	2	2

FAP1_B03	1	1	1	0	0	0	2	1	1	1	1	1	2	1	2	0	1	1
FAP1_B04	1	0	2	0	2	1	0	1	2	0	2	0	2	0	2	1	0	1
FAP1_B05	1	0	2	0	2	0	1	2	2	1	2	1	1	0	2	1	1	1
FAP1_B06	2	2	1	0	0	1	0	0	1	1	1	0	2	1	2	2	1	2
FAP1_B07	1	0	2	0	1	1	1	0	2	2	0	0	2	1	2	1	1	2
FAP1_B08	0	0	1	0	1	2	1	1	2	0	0	1	1	0	2	1	1	1
FAP1_B10	1	0	2	0	0	2	0	0	1	1	0	1	0	1	2	1	2	2
FAP1_B11	2	1	1	0	0	2	1	0	1	1	0	1	2	0	2	0	0	2
FAP1_C01	0	0	2	0	1	1	0	1	1	1	0	0	2	1	2	0	0	2
FAP1_C03	1	0	1	1	1	2	2	2	2	1	1	1	2	2	0	1	1	0
FAP1_C04	0	0	2	0	2	1	2	1	2	0	0	0	1	1	2	0	1	1
FAP1_C05	0	0	1	0	2	2	2	1	2	1	1	0	2	0	2	0	0	1
FAP1_C07	1	1	2	0	2	1	0	2	2	0	0	2	2	1	2	1	0	1
FAP1_C09	0	0	1	0	1	1	0	2	2	1	0	0	2	1	2	0	0	2
FAP1_C12	1	0	1	0	1	2	0	0	1	1	1	1	2	2	2	2	1	2
FAP1_D01	0	0	1	0	1	2	0	2	2	1	0	0	1	0	2	1	0	1
FAP1_D03	0	0	1	0	1	2	0	0	0	0	0	0	2	1	2	1	0	1
FAP1_D04	1	1	0	0	1	2	1	1	2	1	0	0	2	2	1	1	2	1
FAP1_D05	2	1	2	0	1	1	0	1	1	1	0	0	2	2	2	1	0	2
FAP1_D06	0	1	0	0	2	2	0	0	2	0	0	0	2	1	2	1	1	1
FAP1_D07	0	0	2	0	1	2	0	0	2	2	1	0	1	2	2	1	1	2
FAP1_D08	0	1	2	0	2	2	0	0	1	2	1	1	2	1	2	1	0	1
FAP1_D09	1	1	1	0	1	2	1	1	1	1	0	0	1	1	2	0	0	1
FAP1_D11	0	0	1	0	1	2	2	1	1	1	0	1	2	1	2	0	0	2
FAP1_D12	1	1	2	0	1	2	0	1	1	1	1	1	1	2	2	0	1	2
FAP1_E01	0	0	2	0	0	2	0	1	2	1	0	0	2	2	2	1	0	2
FAP1_E03	0	0	1	0	1	2	2	1	1	2	0	1	2	1	2	0	0	2
FAP1_E04	1	0	2	0	0	2	0	2	2	2	2	2	2	1	2	2	1	1
FAP1_E06	1	0	1	0	2	2	0	0	1	2	1	0	2	2	2	0	1	2
FAP1_E07	2	1	0	0	1	2	0	1	1	1	0	0	0	2	2	2	0	2
FAP1_E08	1	0	0	0	1	2	0	0	2	2	0	0	1	2	2	1	1	1
FAP1_E09	0	0	1	0	1	2	0	2	2	1	0	0	1	0	2	0	1	2
FAP1_E11	1	1	1	0	1	1	0	1	1	1	1	0	2	2	1	1	1	2
FAP1_F01	0	0	2	0	0	1	0	0	2	1	0	0	2	1	2	0	0	2
FAP1_F02	1	0	2	0	1	1	1	0	2	1	0	2	1	1	2	1	2	2
FAP1_F03	2	0	2	0	0	0	1	1	2	1	1	1	1	0	2	2	2	1
FAP1_F04	0	0	2	0	2	1	0	0	1	1	2	2	0	2	2	1	1	1
FAP1_F05	2	1	2	0	1	2	1	0	2	1	1	0	2	1	2	1	1	0
FAP1_F06	0	0	2	0	1	1	2	1	2	0	1	1	1	0	2	0	0	1
FAP1_F07	0	0	2	0	0	1	0	0	1	1	0	0	2	1	2	0	1	2
FAP1_F10	1	0	1	1	2	1	0	1	2	1	1	1	2	0	2	0	0	1
FAP1_F11	1	0	2	0	1	0	0	1	2	1	0	1	2	0	2	1	1	1
FAP1_G01	0	0	2	0	2	1	0	1	2	1	0	0	1	1	2	0	0	2
FAP1_G03	1	2	2	0	1	2	0	0	2	1	0	0	1	1	2	0	0	1
FAP1_G04	1	0	1	0	1	1	0	1	2	1	1	1	2	2	2	0	1	2
FAP1_G05	2	2	2	0	1	1	1	2	2	2	0	0	2	0	2	1	1	2
FAP1_G06	0	0	1	0	0	2	0	1	2	1	1	1	1	0	1	1	1	1
FAP1_G08	1	0	2	0	2	2	2	2	2	0	1	0	2	1	2	0	1	0
FAP1_G09	2	2	1	0	2	1	2	1	2	0	1	1	2	1	2	0	1	0

FAP1_G11	1	1	2	0	2	2	0	1	1	1	0	0	2	2	1	0	0	0
FAP1_H01	2	2	2	1	0	2	1	1	0	0	0	1	0	1	2	1	0	2
FAP1_H02	1	2	2	0	0	1	0	1	2	1	0	0	1	1	1	0	1	1
FAP1_H03	0	0	1	0	0	2	0	1	1	0	0	0	1	2	1	0	1	0
FAP1_H04	0	0	2	0	2	2	1	1	2	0	2	0	1	0	2	0	0	1
FAP1_H05	1	1	1	0	0	1	0	1	1	2	0	1	1	0	2	1	0	2
FAP1_H06	1	1	2	0	1	2	0	1	1	1	1	0	1	0	2	0	1	2
FAP1_H07	0	0	0	0	0	1	0	0	2	1	1	0	1	1	2	1	1	1
FAP1_H08	0	0	0	0	0	1	0	0	2	1	1	0	1	1	2	1	1	1
FAP1_H10	0	0	0	0	2	2	1	0	0	0	1	1	1	0	1	1	1	1
FAP1_H11	0	1	2	0	0	1	1	1	0	0	1	0	1	1	2	0	0	1
FAP2_A02	1	1	2	0	1	0	0	1	2	0	1	1	1	2	0	1	1	2
FAP2_A03	2	1	1	0	2	2	1	1	2	1	2	0	2	1	0	0	0	1
FAP2_A05	1	1	2	0	1	2	2	1	2	2	2	2	1	1	0	1	1	1
FAP2_A06	2	0	1	0	2	1	0	0	1	0	1	0	2	2	0	0	0	0
FAP2_A08	2	0	1	0	1	1	1	1	2	0	1	1	2	1	1	0	0	2
FAP2_A10	2	0	1	0	1	2	0	1	2	1	2	1	2	0	1	1	0	1
FAP2_A11	1	0	2	0	1	2	0	2	1	0	1	0	1	1	0	0	1	2
FAP2_A12	1	0	2	0	1	1	1	1	2	1	1	0	2	1	1	1	0	2
FAP2_B01	1	1	2	0	2	2	0	1	1	1	0	0	2	2	1	0	0	0
FAP2_B02	0	0	2	0	2	2	0	1	1	2	1	0	2	1	2	1	2	0
FAP2_B03	0	0	2	0	2	1	0	1	1	1	2	0	1	2	2	2	0	0
FAP2_B04	0	0	2	0	2	1	1	0	1	1	0	0	2	0	2	0	0	2
FAP2_B05	0	0	2	0	1	2	1	2	2	1	2	0	1	1	1	1	0	0
FAP2_B06	2	1	2	0	1	2	0	2	2	2	0	0	2	1	2	0	1	2
FAP2_B08	0	0	1	0	0	1	1	1	2	1	0	0	2	0	2	0	0	2
FAP2_B09	0	0	2	0	2	2	0	1	2	2	1	0	2	1	2	1	1	2
FAP2_B10	2	1	2	0	0	2	0	0	0	0	1	0	2	0	0	1	1	1
FAP2_B11	1	1	2	0	0	2	1	0	2	1	1	1	1	1	2	0	1	0
FAP2_B12	0	0	0	0	1	2	1	0	1	1	0	0	1	1	2	1	1	1
FAP2_C01	1	1	2	0	2	1	1	1	0	0	1	1	2	1	1	0	1	0
FAP2_C02	1	0	2	0	2	0	1	1	2	2	1	2	2	2	2	1	0	1
FAP2_C04	1	0	2	0	2	2	0	2	2	1	1	0	1	2	2	1	0	0
FAP2_C05	0	0	2	1	1	2	1	0	0	0	1	1	0	1	2	1	0	2
FAP2_C06	0	0	2	0	1	2	0	1	2	0	1	0	2	2	2	1	1	1
FAP2_C07	0	1	2	0	1	1	0	1	0	1	0	1	2	2	2	2	1	2
FAP2_C08	1	1	1	0	0	0	2	0	2	0	0	0	2	1	1	1	1	2
FAP2_C09	1	1	1	1	1	1	2	1	1	1	1	1	2	0	2	1	0	2
FAP2_C10	0	0	1	0	1	0	0	0	1	1	1	0	2	2	2	1	1	1
FAP2_C11	1	1	2	1	2	2	0	0	0	1	2	1	1	2	2	0	1	1
FAP2_C12	1	0	1	1	0	1	2	1	1	1	1	0	0	2	2	0	1	0
FAP2_D01	0	1	1	0	0	0	0	0	1	1	2	0	0	0	2	0	0	1
FAP2_D02	0	0	1	1	0	2	0	1	1	2	0	0	1	0	1	1	2	2
FAP2_D03	2	0	1	0	1	0	0	1	2	0	1	2	1	1	2	1	0	2
FAP2_D04	0	0	2	0	1	2	2	0	2	0	0	0	1	1	2	1	0	0
FAP2_D05	0	0	2	0	1	1	1	0	1	1	0	1	0	2	2	1	2	2
FAP2_D06	2	2	2	0	1	2	2	2	2	0	1	1	1	0	2	1	2	1
FAP2_D07	0	1	2	0	2	2	0	1	1	0	2	1	2	1	2	1	1	2
FAP2_D08	2	0	2	0	1	0	1	0	0	0	2	0	1	1	2	0	1	1

FAP2_D09	0	0	2	0	2	1	0	0	0	0	0	1	2	1	2	1	1	1
FAP2_D10	1	0	1	0	0	2	1	1	2	1	0	0	1	0	2	2	1	0
FAP2_D11	1	1	2	0	1	1	0	1	1	1	2	1	2	0	2	2	1	2
FAP2_D12	1	1	2	1	1	2	1	0	2	1	2	0	0	1	2	0	1	0
FAP2_E01	0	0	0	0	0	1	1	1	2	1	0	0	2	1	2	1	0	2
FAP2_E03	1	1	2	0	0	1	2	2	2	0	0	0	1	1	2	0	0	0
FAP2_E04	1	1	2	0	1	1	0	1	2	1	1	0	1	1	2	0	0	0
FAP2_E05	1	0	0	0	1	2	0	0	2	2	0	0	1	2	2	1	1	1
FAP2_E08	0	0	1	0	0	2	0	1	2	0	2	1	2	2	2	0	0	0
FAP2_E09	1	1	2	0	1	1	0	1	2	1	2	1	2	2	2	1	0	2
FAP2_E11	1	0	2	1	1	1	1	2	2	1	0	2	0	1	2	0	1	2
FAP2_F02	1	0	2	1	1	1	1	2	2	1	0	2	0	1	2	1	1	2
FAP2_F03	2	0	1	1	1	1	0	1	2	1	0	1	0	2	2	1	2	2
FAP2_F05	0	0	2	0	1	1	0	1	2	1	0	0	1	1	2	0	2	1
FAP2_F07	1	1	0	0	2	2	1	0	0	1	0	0	2	0	2	1	1	0
FAP2_F08	0	0	0	0	2	2	1	1	1	2	0	0	2	0	2	1	0	2
FAP2_F09	0	0	2	0	1	1	0	1	2	1	0	0	1	1	2	0	1	1
FAP2_F10	0	0	2	0	2	1	0	2	0	1	0	0	2	0	2	1	1	1
FAP2_F11	1	1	2	0	2	1	2	1	1	1	0	0	2	2	2	0	1	2
FAP2_F12	0	2	1	0	1	2	0	0	2	1	0	0	1	1	2	0	0	1
FAP2_G01	1	1	2	0	1	1	1	1	2	1	0	1	2	1	2	1	1	1
FAP2_G02	1	1	2	0	2	1	0	2	2	0	0	2	2	1	2	1	0	0
FAP2_G03	1	1	1	0	0	1	0	1	1	2	0	1	1	0	2	1	1	2
FAP2_G04	2	0	2	0	2	1	0	0	2	1	1	0	1	1	2	0	0	2
FAP2_G05	1	1	2	0	2	2	0	1	1	1	0	0	2	2	1	0	0	0
FAP2_G06	2	1	2	0	1	2	1	0	2	1	1	0	2	1	2	1	1	0
FAP2_G07	2	2	2	0	1	2	2	2	2	0	1	1	1	0	2	1	1	2
FAP2_G08	1	0	1	0	2	1	1	1	2	1	0	1	2	0	2	1	0	1
FAP2_G09	1	0	1	0	1	1	0	1	2	1	1	1	2	2	0	0	1	2
FAP2_G10	2	0	1	0	1	1	1	1	2	1	1	1	2	1	1	0	0	2
FAP2_G11	0	0	2	0	1	2	0	0	2	1	1	0	1	2	2	1	1	2

Supplementary Figure 1: Distribution of adenoma numbers in the 178 multiple adenoma samples

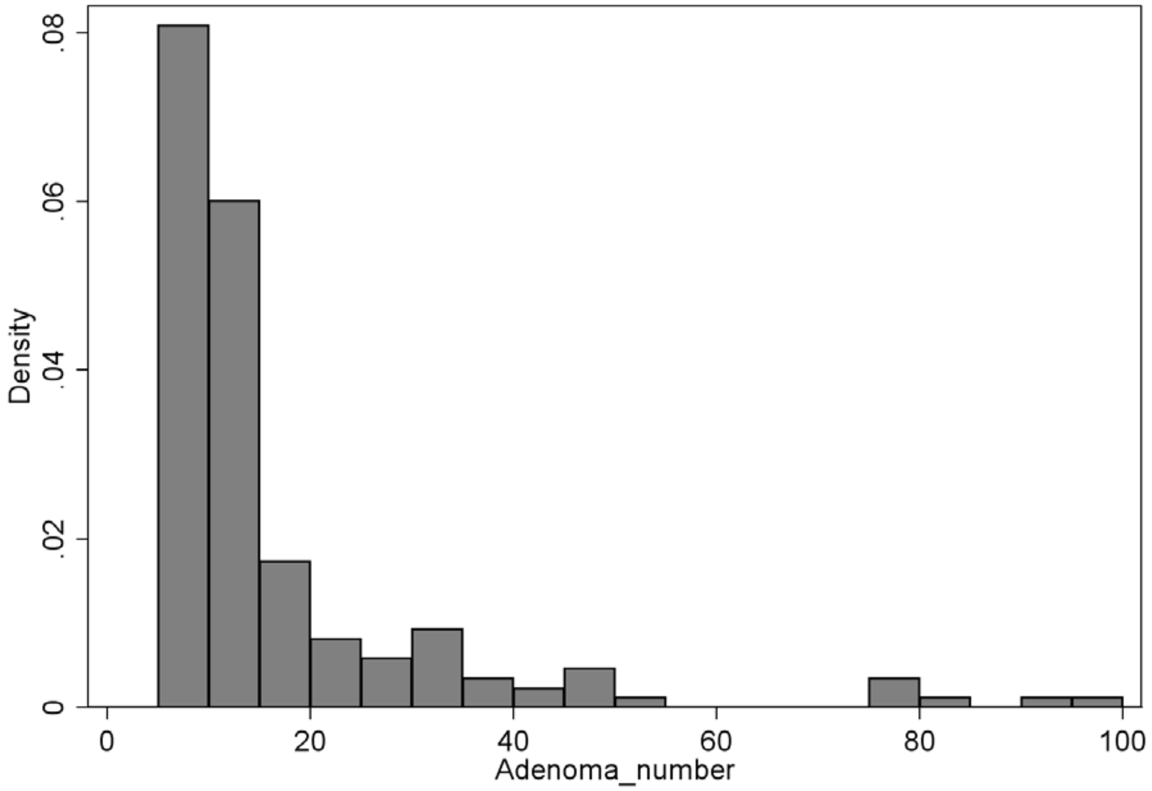

Supplementary Figure 2: Principal component analysis (PCA)

PCA was performed using 40,000 independent SNPs on US controls of northern European origin from the colorectal CFR and CGEMS studies, CRC cases from the CFR and individuals from the same UK population as the multiple adenoma cases (white, European CORGI study cases and controls – see Tomlinson et al, Br J Cancer;102: 447-454). We initially excluded several hundred individuals from the USA who did not cluster within the main centroids of Eigenvectors 1-6. We then included the remaining CFR controls in the study as controls for the multiple adenoma cases. The CFR cases and CGEMS controls were used only to provide unbiased effect size estimates for the risk score calculations (Supplementary Table 1). The plot shows the first two Eigenvectors after sample exclusion, and demonstrates that the US samples used for our analysis cluster well with UK individuals. Subsequent Eigenvectors show very similar results.

.

.

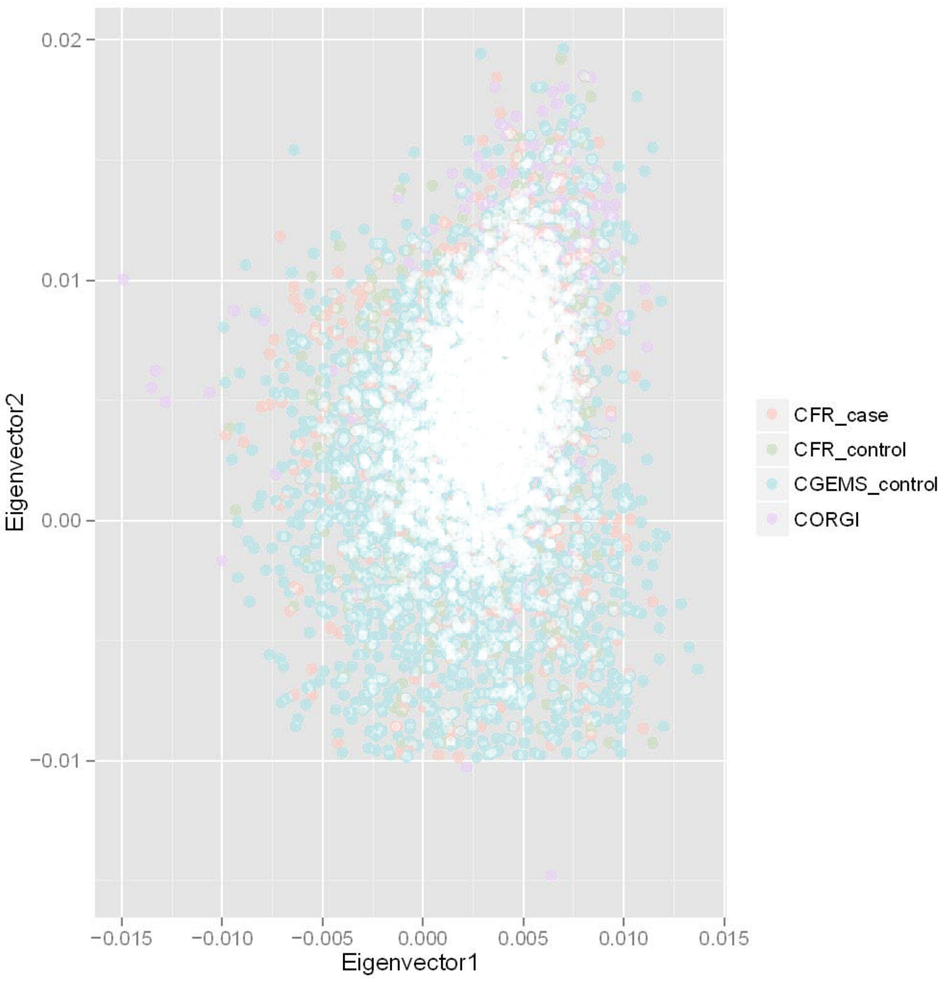

Supplement: Supplementary Information [file ejhg201474x1.pdf]
